# Efficient Communications in V2V Networks with Two-Way Lanes Based on Random Linear Network Coding

**DOI:** 10.3390/e25101454

**Published:** 2023-10-17

**Authors:** Yiqian Zhang, Tiantian Zhu, Congduan Li

**Affiliations:** 1 School of Electronics and Communication Engineering, Sun Yat-sen University, Shenzhen 518107, China; zhangyq75@mail2.sysu.edu.cn (Y.Z.); 18705168317@163.com (T.Z.); 2 Shenzhen Key Laboratory of Navigation and Communication Integration, Shenzhen 518107, China

**Keywords:** random linear network coding, vehicle-to-vehicle communication, dynamic topology, latency reduction

## Abstract

Vehicle-to-vehicle (V2V) communication has gained significant attention in the field of intelligent transportation systems. In this paper, we focus on communication scenarios involving vehicles moving in the same and opposite directions. Specifically, we model a V2V network as a dynamic multi-source single-sink network with two-way lanes. To address rapid changes in network topology, we employ random linear network coding (RLNC), which eliminates the need for knowledge of the network topology. We begin by deriving the lower bound for the generation probability. Through simulations, we analyzed the probability distribution and cumulative probability distribution of latency under varying packet loss rates and batch sizes. Our results demonstrated that our RLNC scheme significantly reduced the communication latency, even under challenging channel conditions, when compared to the non-coding case.

## 1. Introduction

Vehicles serve as vital means of transportation in urban cities, necessitating increased intelligence, as the intelligence of a single car falls short of meeting the requirements for road safety, path planning, decision-making, and traffic efficiency. To address these challenges, the Internet of Vehicles (IoV) has been introduced, enabling communication and collaboration among vehicles, and playing a crucial role in slow-vehicle warnings, intersection collision warnings [1], as well as congestion alleviation, emission reduction, and time saving [2]. However, these networks face obstacles in terms of mobility and occlusion. Notably, when a vehicle is traveling at a speed of 120 km/h, a mere 1-second latency can result in a driving distance of 33 m, potentially leading to severe consequences. Initially, communication among vehicles relied on dedicated short-range communication (DSRC) technology [3], which facilitated short-range communication with low latency [4]. Nevertheless, in high-speed scenarios, vehicles often move out of the communication range, rendering DSRC inadequate. The advent of 5G technology has introduced the concept of ultra-reliable and low-latency communications (uRLLC) [5], enabling vehicles to maintain communication even during high-speed mobility scenarios. To minimize long-term content access costs in vehicle-to-vehicle (V2V) networks, ref. [6] considered a distributed multi-agent reinforcement learning (MARL)-based edge caching method and proposed a distributed MARL-based edge caching method (DMRE), where every agent adaptively learns optimal caching strategies in collaboration with others. Additionally, they integrated the advantages of deep Q-Networks into DMRE, resulting in a computationally efficient method named DeepDMRE, which utilizes neural networks to approximate Nash equilibria. Such deep Q-Networks were also considered in [7], to explore the integration of reconfigurable intelligent surfaces (RIS) with unmanned aerial vehicles (UAVs) in the downlink of non-orthogonal multiple-access (NOMA) networks. They proposed a joint optimization scheme using deep Q-networks to maximize system capacity, while considering UAV energy constraints and demonstrating significant improvements in system capacity.

Network coding [8] offers a promising solution for enhancing the performance of communication systems in V2V networks. By employing network coding techniques, intermediate nodes in the network can encode the received messages before transmitting them to the next hop, and the sink node decodes the received messages to reconstruct the original information. Ref. [9] proposed the use of XOR network coding in fault-tolerant dynamic scheduling and routing algorithms for time-sensitive in-vehicle networks (IVNs), to increase throughput, reliability, and robustness. Experimental results demonstrated that the XOR network coding scheme outperformed the frame replication and elimination for reliability (FRER) mechanism in terms of schedulability, flow, and response time, because the FRER mechanism tends to over-utilize the available bandwidth, whereas XOR network coding provides a better performance without excessive bandwidth usage. Ref. [10] expanded upon the security and privacy considerations in V2V networks as the number of vehicles accessing the network increases and proposed a comprehensive scheme that combines network coding, relay collaboration, and homomorphic encryption. The scheme ensures that the original information remains inaccessible to relay nodes, except for the intended target vehicle node. It also protects against potential collusion attacks, preventing conspiratorial attackers or multiple relay nodes from recovering the original information. Theoretically, such schemes guarantee the confidentiality, privacy protection, and anti-collusion capabilities of V2V networks. In [11], F. Ye et al. adopted network coding in vehicular ad hoc networks (VANETs) by modeling platoon vehicles driving in the same direction on a highway as a 1-D lattice network, in which a single source node aims to disseminate messages to all other vehicles. They analyzed the theoretical upper bound of the benefits achieved through network coding and conducted simulations to demonstrate the performance superiority over random broadcasting using Rayleigh fading wireless channels. F. Liu et al. [12] extended the data dissemination in VANETs in [11] to a two-way lane scenario by modeling the network as two separate 1-D lattice networks, corresponding to the two directions of traffic flow. They divided the dissemination into the *encountering* phase and the *separated* phase, determined by whether the broadcasting coverage areas of the two disseminators overlapped, which means vehicles traveling in both direction can communicate with both disseminators simultaneously. They analyzed the impact of the opposite direction over the traditional one-way lane model and showed that two disseminators traveling in opposite directions can enhance the speed of data dissemination. Ref. [13] compared three methods in a highway data mulling scenario, with vehicles from the opposite direction as data mutes to transmit large multimedia files, modeled as a *coupon collector* problem, and among which the network-coding-based strategy outperformed erasure-coding and repetition-coding strategies. The literature [14,15,16] shows that network coding can improve reliability and throughput, but it fails to deal with dynamic situations where the vehicle volume increases rapidly and the network structure becomes complex. Therefore, random linear network coding (RLNC) [17] has garnered significant attention, particularly for its ability to operate without prior knowledge of the network topology. RLNC involves random coding coefficient selection from a finite field and performing linear operations on the packets. As the vehicular scale increases, the random selection of RLNC encoding coefficients within a finite field obviates the need to account for variations in node quantity and network topology within this method. By receiving a sufficient number of packets with independent coefficients at the sink, the original information can be decoded at source, which enables transmitting content over wireless vehicle communications with lossy links and that are highly dynamic. Ref. [18] proposed a RLNC scheme for data transmission in a one-way lane V2V network, modeled as a multi-source multi-relay single-sink broadcasting network, to reduce latency and enhance the network robustness. In this one-way lane V2V communication scenario, the leading vehicles relay the detected road conditions and critical safety alerts to those following behind, affording them sufficient time for well-informed decision-making. This type of information, with its small data payload, facilitates swift transmission with no node departures in multi-round communication processes, as assumed in [18], and implies a static and unchanging network topology. This may not align with the evolving landscape of intelligent transportation. In particular, with the increasing demand for in-vehicle entertainment experiences, expediting the transmission of large-scale data from nearby vehicles has become essential. Given the significant data volume involved, this study explores the utilization of vehicles in the opposite lane to establish a framework for bidirectional V2V large-scale data transmission over an extended period. During prolonged communication sessions for large-scale data transmission, nodes at high speeds tend to exit the communicable range of receiving vehicles, leading to dynamic changes in the network topology over multiple rounds. In this extended two-way lane large-scale data transmission scenario, the network is modeled as a multi-source single-sink network with a dynamic topology, where cars may enter or leave the communication range, resulting in a varying number of sources each round. The destination car node receives information from cars traveling in both the same and opposite directions. By utilizing RLNC in this dynamic two-way lane model, the proposed scheme enhances throughput and robustness, without relying on a specific network topology.

The main contributions of this paper are as follows:

- it extends the one-way lane model proposed in [18] to incorporate two-way lanes, thereby creating a dynamic network model;
- the paper provides a lower bound on the generation probability, demonstrating the feasibility and effectiveness of the RLNC scheme;
- it evaluates the performance of the RLNC scheme under frequently changing network conditions and poor channel conditions. The results demonstrate that RLNC significantly reduces latency compared to non-coding schemes.

The rest of this paper is organized as follows: Section 2 provides a brief overview of RLNC and compares our work with the related literature. Section 3 presents a system model, detailing the two-way lane RLNC transmission scheme and conducting an analysis of the generation probability and time delay. In Section 4, we analyze the simulation performance for communication delays under different packet loss rates and batch sizes, and then compare the coding and non-coding schemes. Section 5 concludes the paper.

## 2. Related Work

In this section, we give details about RLNC and introduce the reasons why RLNC is used. Then, the recent related literature for RLNC in V2V networks is compared with our work.

### 2.1. Brief of RLNC

**Figure 1:**
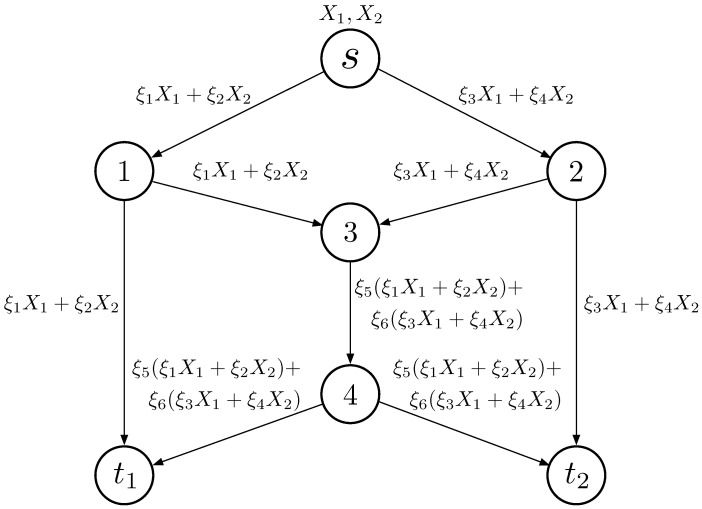
RLNC model in a butterfly network.

We first give a brief introduction to RLNC. Li et al. proposed linear network coding (LNC) in [19], where they allowed intermediate nodes in the network to perform operations on the incoming packets, combining them linearly before forwarding. At the receiving end, the nodes can then decode the received combinations, to retrieve the original information. Ho et al. then proposed LNC in a randomized setting [20], where the coding coefficients are randomly chosen in a fixed-size finite field. Figure 1 illustrates a straightforward application of RLNC in a butterfly network. Source node *s* is tasked with sending messages $X_{1}$ and $X_{2}$ to sinks $t_{1}$ and $t_{2}$. Each channel can transmit only one message during a given time slot. Node *s* sends the linearly encoded $X_{1}$ and $X_{2}$ with the randomly selected coefficients $\left(\xi_{1},\xi_{2}\right)$, resulting in $\xi_{1}X_{1}+\xi_{2}X_{2}$, to node 1. This information is then forwarded to nodes 3 and $t_{1}$. Similar operations occur at node 2, with randomly chosen coefficients $\left(\xi_{3},\xi_{4}\right)$. Given that node 3 receives two messages but can only utilize one channel to communicate with node 4, it becomes imperative to perform linear network coding at node 3 using randomly chosen coefficients $\left(\xi_{5},\xi_{6}\right)$. Subsequently, node 4 forwards the encoded message to both sinks. The messages received at $t_{1}$ are denoted as $Y_{11}$ and $Y_{12}$, which is

(1) $$\left[\begin{array}{c} Y_{11}\\ Y_{12} \end{array}\right]=\left[\begin{array}{cc} \xi_{1} & \xi_{2}\\ \xi_{5}\xi_{1}+\xi_{6}\xi_{3} & \xi_{5}\xi_{2}+\xi_{6}\xi_{4} \end{array}\right]\left[\begin{array}{c} X_{1}\\ X_{2} \end{array}\right],$$

and the messages received at $t_{2}$ are denoted as $Y_{21}$ and $Y_{22}$, which is

(2) $$\left[\begin{array}{c} Y_{21}\\ Y_{22} \end{array}\right]=\left[\begin{array}{cc} \xi_{3} & \xi_{4}\\ \xi_{5}\xi_{1}+\xi_{6}\xi_{3} & \xi_{5}\xi_{2}+\xi_{6}\xi_{4} \end{array}\right]\left[\begin{array}{c} X_{1}\\ X_{2} \end{array}\right],$$

With invertible coefficient matrices, the original $X_{1}$ and $X_{2}$ can be decoded.

### 2.2. RLNC in V2V

Many works have introduced RLNC to V2V communication scenarios. Considering massive gigabit content transmission in millimeter-wave networks, ref. [21] applied symbol-level network coding (SLNC); that is, RLNC at the symbol scale, and utilized a cooperative concurrent distribution strategy in the scenario of highway network topology, where roadside units (RSU) encode the original packets and then forward them to vehicles. The proposed scheme enables collaborative V2V and vehicle-to-infrastructure (V2I) mmWave communications through a greedy network coding strategy based on a graph-theoretic approach. The scheme achieves a low latency, high efficiency, error resilience, and reliability. In ref. [22], E. Tasdemir et al. implemented a dynamic systematic sliding window RLNC scheme for end-to-end communication in vehicle platooning scenarios, where the platooning leader generates packets that are transmitted hop-by-hop to the platooning members. The coding process only involves packets within the dynamically sliding window, which moves forward to include new packets and is closed through feedback, and these packets are combined linearly to generate coded packets using RLNC techniques. This coding scheme was shown through simulation to provide resilience and low latency. To address challenges like transmission collisions and channel fading, ref. [23] proposed a hybrid medium access control (MAC) protocol for basic safety messages (BSMs) dissemination within the DSRC framework. Additionally, this protocol, with three sessions, a MAC setup session, CSMA session, and PNC session integrating physical-layer network coding and RLNC, further enhances the reliability and efficiency of BSM dissemination. Ref. [24] further analyzed packet delivery ratio performance theoretically and through a comprehensive simulation. Our proposed method is compared with the recent literature works in a comparative table, as Table 1.

## 3. System Model and RLNC Algorithm

In this section, we give the system model and introduce the RLNC algorithm.

### 3.1. System Model

**Figure 2:**
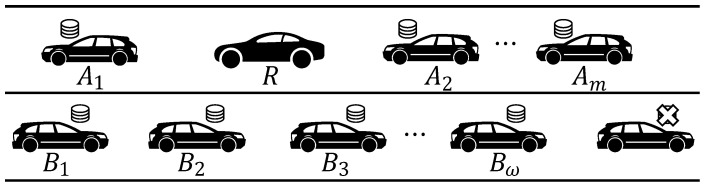
Vehicle road model.

First, we build the two-way lane V2V model based on real vehicle road scenarios, as illustrated in Figure 2. In the model, the car receiving messages, denoted as *R* and travels at a speed of $v_{R}$. We have *m* cars, denoted as $A_{1},A_{2},\cdots ,A_{m}$, traveling in the same direction as *R* at constant speeds of $v_{A_{1}},v_{A_{2}},\cdots ,v_{A_{m}}$, respectively. Additionally, there are *w* cars, denoted as $B_{1},B_{2},\cdots ,B_{w}$, traveling in the opposite direction at constant speeds of $v_{B_{1}},v_{B_{2}},\cdots ,v_{B_{w}}$, respectively. For each i∈1,2,⋯,m and j∈1,2,⋯,w, cars $A_{i}$ and $B_{j}$ store *M* identical raw packets to be transmitted. These *M* raw packets collectively form a *generation*. Once the sink node *R* receives (or decodes) all *M* raw packets, the raw packets are updated to transmit the next generation.

*R* only communicates with cars within its communication range *d*. Specifically, *R* and $A_{i}$ establish contact only when the distance between them, denoted as $d_{\left(A_{i},R\right)}$, satisfies $d_{\left(A_{i},R\right)}<d$. In the case where $A_{i}$ is positioned ahead of *R*, the communication between *R* and $A_{i}$ can be maintained for

(3) $$t=\frac{d+d_{\left(A_{i},R\right)}}{v_{R}-v_{A_{i}}};$$

When $v_{A_{i}}>v_{R}$, the communication between *R* and $A_{i}$ can be maintained for

(4) $$t=\frac{d-d_{\left(A_{i},R\right)}}{v_{A_{i}}-v_{R}}.$$

If $v_{A_{i}}=v_{R}$, they can always communicate with each other.

In addition, when $A_{i}$ is positioned behind *R*, when $v_{A_{i}}<v_{R}$, the communication between *R* and $A_{i}$ can be maintained for

(5) $$t=\frac{d-d_{\left(A_{i},R\right)}}{v_{R}-v_{A_{i}}};$$

When $v_{A_{i}}>v_{R}$, the communication between *R* and $A_{i}$ can be maintained for

(6) $$t=\frac{d+d_{\left(A_{i},R\right)}}{v_{A_{i}}-v_{R}}.$$

If $v_{A_{i}}=v_{R}$, they can always communicate with each other.

Regarding the opposite lane, if car $B_{i}$ is moving towards *R*, then

(7) $$t=\frac{d-d_{\left(B_{i},R\right)}}{v_{R}+v_{B_{i}}};$$

If car $B_{i}$ is traveling in the opposite direction and is positioned behind car *R*, *R* and $B_{i}$ can keep in touch for

(8) $$t=\frac{d+d_{\left(B_{i},R\right)}}{v_{R}+v_{B_{i}}}.$$

**Figure 3:**
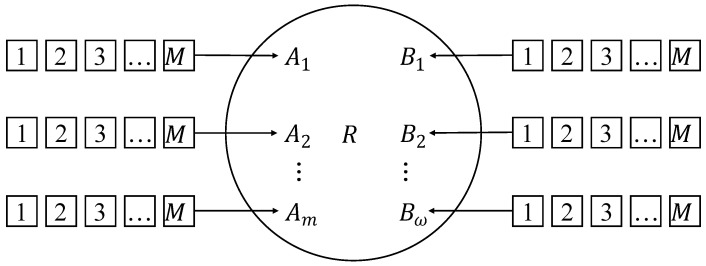
A sketch of a two-way lane model.

We further extract the model as a multi-source single-sink network, as shown in Figure 3. In this model, the sink node is denoted *R*. The cars traveling in the same direction are $A_{\alpha_{t}}$, where $\alpha_{t}=0,1,2,\ldots ,m$. Similarly, the cars traveling in the opposite direction are denoted as $B_{\beta_{t}}$, with $\beta_{t}=0,1,2,\ldots ,w$. Both the cars in the same direction and those in the opposite direction possess identical sets of *M* raw data packets, collectively referred to as a generation. These packets are organized into batches to be transmitted. It is important to note that the number of source nodes, denoted by $\alpha_{t}$ and $\beta_{t}$, may vary in each round.

### 3.2. RLNC Algorithm

We now implement the RLNC algorithm, analyze the probability of generation, and present the corresponding algorithms. To implement RLNC, we select encoding coefficients from the finite field GF(q), where the size of the finite field is denoted *q*. Consequently, we obtain the encoded data packet $\Gamma_{A_{i}}$ transmitted by the source node $A_{i}$ from the same direction as

(9) $$\Gamma_{A_{i}}=a_{i}^{1}r_{1}+a_{i}^{2}r_{2}+a_{i}^{3}r_{3}+\cdots +a_{i}^{M}r_{M},$$

where $r_{k}$ represents the *k*th data packet, and $a_{i}^{k}\in GF\left(q\right)$ denotes the encoding coefficient associated with $r_{k}$ in the encoded data packet $\Gamma_{A_{i}}$. Here, *i* ranges from 0 to $\alpha_{t}$ (the number of source nodes in the same direction), and *k* ranges from 1 to *M* (the total number of data packets in a generation). Similarly, the encoded data packet $\Gamma_{B_{j}}$ from the opposite source $B_{j}$ is

(10) $$\Gamma_{B_{j}}=b_{j}^{1}r_{1}+b_{j}^{2}r_{2}+b_{j}^{3}r_{3}+\cdots +b_{j}^{M}r_{M},$$

where $b_{j}^{k}\in GF\left(q\right)$ is the encoding coefficient of $r_{k}$ in $\Gamma_{B_{i}}$, $j=0,1,2,\ldots ,\beta_{t}$, k=1,2,…,M.

In each time slot, the source nodes collectively transmit the $\alpha_{t}+\beta_{t}$ packets that have been encoded using RLNC, which can be represented in matrix form as follows:

(11) $$\begin{array}{cc} \left[\begin{array}{c} \Gamma_{A_{1}}\\ \Gamma_{A_{2}}\\ \cdots \\ \Gamma_{A_{\alpha_{t}}}\\ \Gamma_{B_{1}}\\ \Gamma_{B_{2}}\\ \cdots \\ \Gamma_{B_{\beta_{t}}} \end{array}\right]\; & =\left[\begin{array}{cccc} a_{2}^{1} & a_{2}^{2} & \cdots & a_{2}^{M}\\ a_{1}^{1} & a_{1}^{2} & \cdots & a_{1}^{M}\\ \cdots & \; & & \cdots \\ a_{\alpha_{t}}^{1} & a_{\alpha_{t}}^{3} & \cdots & a_{\alpha_{t}}^{M}\\ b_{1}^{1} & b_{1}^{2} & \cdots & b_{1}^{M}\\ b_{2}^{1} & b_{2}^{2} & \cdots & b_{2}^{M}\\ \cdots & \; & & \cdots \\ b_{\beta_{t}}^{1} & b_{\beta_{t}}^{2} & \cdots & b_{\beta_{t}}^{M} \end{array}\right]\left[\begin{array}{c} r_{1}\\ r_{2}\\ r_{3}\\ \cdots \\ r_{M-1}\\ r_{M} \end{array}\right] \end{array}$$

(12) $$\begin{array}{cc} \; & =C_{(\alpha_{t}+\beta_{t})\times M}R_{M\times 1}, \end{array}$$

where $C_{(\alpha_{t}+\beta_{t})\times M}$ is the coefficient matrix and $R_{M\times 1}$ is the raw packet matrix.

#### 3.2.1. Generation Probability

In order to decode *M* raw packets, the sink node *R* needs to receive *M* linearly independent encoded packets. If the encoding coefficient vector of a packet is linearly independent of the encoding data packets previously received, then this packet contributes to the decoding process. We define the number of linearly independent packets received by the sink node as the “sink’s state”, denoted as $S_{R}$. The generation probability, which represents the probability that the encoding packets are linearly independent, takes different forms based on the sink’s state. Specifically, it depends on whether $S_{R}$ is greater than or less than $M-\alpha_{t}-\beta_{t}$. To address these scenarios, we give Lemmas 1 and 2.

**Lemma** **1.** *When the sink’s state $S_{R}\leq M-\alpha_{t}-\beta_{t}$, generation probability is of the form*

(13) $$P_{ge=\alpha_{t}+\beta_{t}}=\prod_{l=i}^{i+\alpha_{t}+\beta_{t}-1}\left(1-\frac{1}{q^{M-l}}\right),$$

*where q is the Galois field size, M is the number of raw packets in each batch, and i is the current state of the sink node, $\alpha_{t}=0,1,2,\cdots ,m$, $\beta_{t}=0,1,2,\cdots ,w$, $i=0,1,2,\cdots ,M-\alpha_{t}-\beta_{t}.$*

**Proof.**  Similarly to the proof of Theorem 1 in [18] but with $n=\alpha_{t}+\beta_{t}$, source nodes send $\alpha_{t}+\beta_{t}$ data packets in a time slot and the *n* packets are regarded as a group. *n* sources send *n* data packets $\Gamma_{\Upsilon_{\eta }}$ in one time slot. There are $q_{M}-1$ choices, resulting in

(14) $$C_{\left(q^{M}-1\right)+\alpha_{t}+\beta_{t}-1}^{\alpha_{t}+\beta_{t}}=C_{q^{M}+\alpha_{t}+\beta_{t}-2}^{\alpha_{t}+\beta_{t}}$$

kinds of combination.

If all the *n* packets are linearly independent, then there are

(15) $$\frac{\prod_{j=0}^{\alpha_{t}+\beta_{t}-1}C_{q^{M}-q^{i+j}}^{1}}{A_{\alpha_{t}+\beta_{t}}^{\alpha_{t}+\beta_{t}}}$$

kinds of combination.

Therefore, the generation probability is

(16) $$P_{ge=\alpha_{t}+\beta_{t}}=\frac{\frac{\prod_{j=0}^{\alpha_{t}+\beta_{t}-1}C_{q^{M}-q^{i+j}}^{1}}{A_{\alpha_{t}+\beta_{t}}^{\alpha_{t}+\beta_{t}}}}{C_{q^{M}+\alpha_{t}+\beta_{t}-2}^{\alpha_{t}+\beta_{t}}}=\frac{\prod_{j=0}^{\alpha_{t}+\beta_{t}-1}C_{q^{M}-q^{i+j}}^{1}}{A_{q^{M}+\alpha_{t}+\beta_{t}-2}^{\alpha_{t}+\beta_{t}}}.$$

After simplifying, we can prove this.    □

**Lemma** **2.** *When the sink’s state is $M>S_{R}>M-\alpha_{t}-\beta_{t}$, that is, $S_{R}=M-\alpha_{t}-\beta_{t}+1,M-\alpha_{t}-\beta_{t}+2,\ldots ,M-1$, the generation probability is of the form:*

(17) $$P_{ge=M-i}=\prod_{l=i}^{M-1}\left(1-\frac{1}{q^{M-l}}\right),$$

*where q is the Galois field size, M is the number of raw packets in each batch, and i is the current sink’s state, $i=M-\alpha_{t}-\beta_{t}+1,M-\alpha_{t}-\beta_{t}+2,\ldots ,M-1.$*

**Proof.**  Similarly to the proof of Theorem 2 in [18] but with $n=\alpha_{t}+\beta_{t}$, the generation probability is

(18) $$P_{ge=M-i}=\frac{\frac{\prod_{j=0}^{M-i-1}C_{q^{M}-q^{i+j}}^{1}}{A_{M-i}^{M-i}}}{C_{q^{M}+M-i-2}^{M-i}}=\frac{\prod_{j=0}^{M-i-1}C_{q^{M}-q^{i+j}}^{1}}{A_{q^{M}+M-i-2}^{M-i}}.$$

After simplifying, we can prove this.    □

The generation probability in this context exhibits similarities to the generation probability discussed in [18]. However, in the context of dynamic topology, the generation probability in each round is influenced, not only by the sink’s state, but also by the number of currently communicable source nodes. Specifically, the sink’s state in time slot *t* can be expressed as $S_{R}>M-\alpha_{t}-\beta_{t}$, indicating that the generation probability aligns with the conditions stated in Lemma 2. Conversely, prior to the initiation of the (t+1)th round of communication, due to multiple vehicles departing the communicable range, in time slot t+1, we have $S_{R}\leq M-\alpha_{t+1}-\beta_{t+1}$. In such cases, the generation probability adheres to the conditions specified in Lemma 1. This distinction arises from the changes in the sink’s state and the varying number of communicable source nodes as a consequence of the dynamic topology in the network.

We focus on determining the lower bound of the generation probability. The lower bound is acquired when the sink’s state is M−m−w and sources send m+w data packets. According to Appendix B in [18], let n=m+w, and we establish the lower bound of generation probability

(19) $$\min P_{ge}=\prod_{l=M-m-w}^{M-1}\left(1-\frac{1}{q^{M-l}}\right),$$

which is equivalent to

(20) $$\min P_{ge}=\prod_{\mu =1}^{m+w}\left(1-\frac{1}{q^{\mu }}\right),$$

Thus, the lower bound of the generation probability depends on the total number of sources at the beginning of communication m+w and the Galois field size *q*.

**Figure 4:**
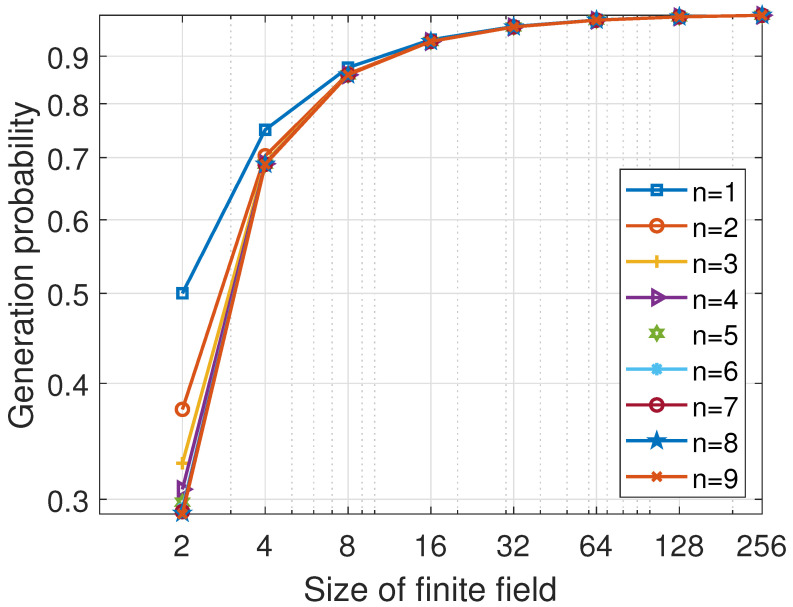
Lower bound of the generation probability with different sources.

Figure 4 illustrates the lower bound of $P_{ge}$, as given by Equation (20), where *n* represents the total number of sources (n=m+w). As the finite field size increases, the lower bound of generation probability also increases. For example, when considering a finite field size of GF(256), the minimum generation probability exceeds 0.996. Consequently, with a sufficiently large finite field size, it is reasonable to assume that every packet transmitted to the sink is valid, and the generation probability approaches 1. Assuming that, after ζ rounds of communication, the sink node has received *M* data packets, the decoding probability can be expressed as

(21) $$P_{d}>\prod_{\mu =1}^{m+w}{\left(1-\frac{1}{q^{\mu }}\right)}^{\zeta }.$$

#### 3.2.2. Time Delay Analysis

In our analysis of the time delay, we consider the dynamic nature of the participating sources in each round, which differs from the one-way lane scenario described in [18]. To address a two-way lane scenario, we first determine the number of source nodes within the communication range of the sink during each round. This is performed based on the position, speed, and initial distance to the sink, as outlined in Algorithm 1. In Algorithm 1, we utilize an indicator variable f. When f=1, this indicates that the source node is initially positioned ahead of the sink node. Conversely, when f=2, this indicates that the source node is initially located behind the sink node. The algorithm utilizes this indicator to determine the number of source nodes present in each round, considering their relative positions with respect to the sink node. We tally the number of same direction sources engaged in each communication round. This is contingent upon whether the source is positioned ahead or behind the destination, as well as the relative speeds of the source and destination vehicles, and the relative distance between them. As for the count of counter-directional sources, this hinges on whether the source is located ahead or behind the destination, along with the relative distance between them.

Based on the number of sources participating in each round, $\alpha_{t}+\beta_{t}$, we obtain the binomial distribution for the state transition of the sink node. When the sink’s state is $S_{R}=0,1,2,\ldots ,M-\alpha_{t}-\beta_{t}$, $\alpha_{t}+\beta_{t}$ source nodes collectively send $\alpha_{t}+\beta_{t}$ valid data packets. The probability of the sink node receiving *k* valid data packets in this round, which corresponds to a transition to *k* states in time slot *t*, can be calculated as

(22) $$P_{mov}\left(t,k\right)=C_{\alpha_{t}+\beta_{t}}^{k}{\left(1-p_{e}\right)}^{k}p_{e}^{\alpha_{t}+\beta_{t}-k},$$

where $p_{e}$ denotes the packet loss rate.
**Algorithm 1** # of source nodes within communication range in time slot *t***Input:** # of source nodes from the same direction *m*    # of source nodes from the reverse direction *w*    communication range $d_{b}$    speed of sink $v_{b}$    position of source nodes in the same direction f, speed v, distance d    position of source nodes in the reverse direction fo, speed vo, distance do    rounds of communication *N*  1:Initialize # of same direction nodes Q=0  2:Q(0)=m  3:**for** i=1→N **do**  4:   count=0  5:   **for** j=0→m−1 **do**  6:     **if** $f\left(j\right)=1\&\&v\left(j\right)<v_{b}\&\&j\left(v_{b}-v\left(j\right)\right)-\left(d_{b}+d\left(j\right)\right)>0$ **then**  7:        count = count − 1  8:     **end if**  9:     **if** $f\left(j\right)=1\&\&v\left(j\right)>v_{b}\&\&j\left(v\left(j\right)-v_{b}\right)-\left(d_{b}-d\left(j\right)\right)>0$ **then**10:        count = count − 111:     **end if**12:     **if** $f\left(j\right)=2\&\&v\left(j\right)<v_{b}\&\&j\left(v_{b}-v\left(j\right)\right)-\left(d_{b}-d\left(j\right)\right)>0$ **then**13:        count = count − 114:     **end if**15:     **if** $f\left(j\right)=2\&\&v\left(j\right)>v_{b}\&\&j\left(v\left(j\right)-v_{b}\right)-\left(d_{b}+d\left(j\right)\right)>0$ **then**16:        count = count − 117:     **end if**18:   **end for**19:   Q(i)=m + count20:**end for**21:Initialize # of reverse direction nodes Qo=022:Qo(0)=w23:**for** i=1→N **do**24:   count = 025:   **for** j=0→w−1 **do**26:     **if** $\mathrm{fo}\left(j\right)=1\&\&j\left(v_{b}+v\left(j\right)\right)-\left(d_{b}+d\left(j\right)\right)>0$ **then**27:        count = count − 128:     **end if**29:     **if** $f\left(j\right)=2\&\&j\left(v_{b}+v\left(j\right)\right)-\left(d_{b}-d\left(j\right)\right)>0$ **then**30:        count = count − 131:     **end if**32:   **end for**33:   Qo(i)=w + count34:**end for**35:total number of the source nodes Q=Q+Qo**Output:** 
# of source nodes within communication range

When the state of the sink is $S_{R}=M-\alpha_{t}-\beta_{t}+1,M-\alpha_{t}-\beta_{t}+2,\cdots ,M-1$, the $M-S_{R}$ source nodes will send $M-S_{R}$ valid data packets. The probability of sink node transits *k* states in time slot *t* is

(23) $$P_{mov}\left(t,k\right)=C_{M-S_{R}}^{k}{\left(1-p_{e}\right)}^{k}p_{e}^{M-S_{R}-k}.$$

The state matrix of the sink in the first time slot is

(24) $$\begin{array}{cc} S_{1}\; & =[B_{n(1)}\left(0\right)\;B_{n(1)}\left(1\right)\cdots \;B_{n(1)}\left(n\left(1\right)\right)] \end{array}$$

(25) $$\begin{array}{cc} \; & =[P_{0,0}^{1}\;P_{0,1}^{1}\cdots \;P_{0,n(1)}^{1}], \end{array}$$

where the binomial distribution $B_{n(t)}\left(\alpha \right)$ represents the probability of receiving α valid data packets out of the data packets sent by n(t) source nodes in time slot *t*. Here, n(t) represents the total number of source nodes in time slot *t*, which is initially m+w. $P_{i,j}^{k}$ denotes the probability of the sink state transitioning from *i* to *j* in the *k*th time slot. To determine the state matrix of the sink node after time slot *t*, denoted as St, we need to first solve for the state matrix after time slot t−1. The state matrix after time slot *t* is denoted St and is represented by Equation (26).

The probability $P_{t}\left(M\right)$, which represents the sink node receiving *M* valid data packets after *t* time slots, can be calculated by summing the probabilities $P_{i,M}^{t}$ over all possible states *i* in the state matrix St. Mathematically, this can be expressed as $P_{t}\left(M\right)=\sum P_{i,M}^{t}$. The solution for the state matrix $S_{t}$ depends on the state matrix $S_{t-1}$ from the previous time slot and the number of source nodes Q(t) derived from Algorithm 1. Algorithm 2 provides a solution for calculating the probability $P_{t}$, which represents the sink node being in different states after time slot *t*. This probability is dependent on the values of $P_{t-1}$ and Q(t−1). Specifically, $P_{t}\left(M\right)$ represents the completion probability of time slot *t*, which is the probability that sink node receives *M* data packets after *t* time slots. The recursive relationship between $P_{t}$ and $P_{t-1}$ is expressed as $P_{t}=\Phi \left(P_{t-1},B_{Q(t-1)}\right)$, where $B_{Q(t-1)}$ represents the binomial distribution of the number of received packets in time slot *t* when Q(t−1) data packets are sent. Such a solution for the recursive relation is provided in Algorithm 3. In Algorithm 3, the first step is to determine the number of source nodes participating in each round of communication. If this is larger than the needed number of packets *M*, then only *M* nodes will participate in the communication. Otherwise, all the nodes will participate. For each sink state *i*, the probability distribution at time slot *t* is computed by calculating the probability of receiving k=i−j messages correctly after having received *j* messages (see line 19 in Algorithm 3). By utilizing Algorithm 2 and Algorithm 3, we can calculate the completion probability $P_{t}\left(M\right)$ of the sink node after *t* time slots. Subsequently, we will conduct an analysis of the delay probability distribution, taking into consideration varying packet loss rates $p_{e}$ and packet batch sizes *M*.

(26) $$\begin{array}{cc} S_{t} & \;=\;\left[\begin{array}{ccccc} \;B_{n(t)}\left(0\right)\sum P_{\eta ,0}^{t-1}\;\; & B_{n(t)}\left(1\right)\sum P_{\eta ,0}^{t-1}\;\; & B_{n(t)}\left(2\right)\sum P_{\eta ,0}^{t-1} & \;\;\cdots \; & B_{n(t)}\left(n\left(t\right)\right)\sum P_{\eta ,0}^{t-1}\\ B_{n(t)}\left(0\right)\sum P_{\eta ,1}^{t-1}\; & B_{n(t)}\left(1\right)\sum P_{\eta ,1}^{t-1}\;\; & B_{n(t)}\left(2\right)\sum P_{\eta ,1}^{t-1}\;\; & \cdots \; & B_{n(t)}\left(n\left(t\right)\right)\sum P_{\eta ,1}^{t-1}\\ \cdots \\ B_{n(t)}\left(0\right)\sum P_{\eta ,M-2}^{t-1} & B_{n(t)}\left(1\right)\sum P_{\eta ,M-2}^{t-1} & B_{n(t)}\left(2\right)\sum P_{\eta ,M-2}^{t-1} & \;\cdots \; & 0\\ B_{n(t)}\left(0\right)\sum P_{\eta ,M-1}^{t-1} & B_{n(t)}\left(1\right)\sum P_{\eta ,M-1}^{t-1} & 0 & \cdots & 0 \end{array}\right]\\ \; & \;=\;\left[\begin{array}{ccccc} P_{0,0}^{t} & P_{0,1}^{t} & P_{0,2}^{t} & \cdots & P_{0,n}^{t}\\ P_{1,1}^{t} & P_{1,2}^{t} & P_{1,3}^{t} & \cdots & P_{1,1+n}^{t}\\ \cdots \\ P_{M-2,M-2}^{t} & P_{M-2,M-1}^{t} & P_{M-2,M}^{t} & \cdots & 0\\ P_{M-1,M-1}^{t} & P_{M-1,M}^{t} & 0 & \cdots & 0 \end{array}\right]. \end{array}$$

**Algorithm 2** Completion probability of sink at time slot *t*
**Input:** 
# of raw data packets *M*, packet loss rate $p_{e}$, communication times *N*, # of source nodes Q1:

$B_{n}\left(i\right)=C_{n}^{i}{\left(1-p_{e}\right)}^{i}p_{e}^{n-i}$

2:**for** i=0→Q(0) **do**3:   $P_{1}\left(i\right)=B_{Q(0)}\left(i\right)$4:
**end for**
5:**for** t=2→N **do**6:   $P_{t}=\Phi \left(P_{t-1},B_{Q_{\left(t-1\right)}}\right)$7:
**end for**
**Output:** 
Completion probability of sink at time slot *t*: $P_{t}\left(M\right)$


**Algorithm 3** The state distribution probability of sink after time slot *t*:$P_{t}=\Phi \left(P_{t-1},B_{Q(t)}\right)$
**Input:** 
The distribution probability of sink state last time slot $P_{t-1}$,# of current source nodes Q(t−1)  1:sumQ(t)=0  2:**for** i=0→t−1 **do**  3:   sumQ(t) = sumQ(t) + Q(i)  4:
**end for**
  5:**if** sumQ(t) > *M* **then**  6:   maxNum = *M*  7:
**else**
  8:   maxNum = sumQ(t)  9:
**end if**
10:sumQ(t − 1) = 011:
**for** i=0→t−2 **do**
12:   sumQ(t − 1) = sumQ(t − 1) + Q(i)13:
**end for**
14:**for**i=0→ maxNum **do**15:   $P_{t}\left(i\right)=0$16:   **for** j=0→ sumQ(t − 1) **do**17:     **for** k=0→Q(t−1) **do**18:        **if** j+k=i&&j!=M **then**19:          $P_{t}\left(i\right)=P_{t}\left(i\right)+P_{t}\left(j\right)\times B_{Q(t-1)}\left(k\right)$20:        **end if**21:     **end for**22:   **end for**23:
**end for**
**Output:** 
The state probability distribution $P_{t}$ of sink after time slot *t*


## 4. Simulation Performance

In this section, we present a comprehensive analysis of the performance of the proposed scheme through Matlab simulations. Our main focus was on minimizing the latency, which was quantified by the number of time slots required to complete the transmission. We paid particular attention to two key factors that impact the latency: the packet loss rate, and the batch size. In addition, we conducted an analysis on the impact of varying vehicle communication ranges and different arrival rates following a stochastic arrival process, the Poisson process. Furthermore, we compared the coding and non-coding schemes. By comparing their performance, we could evaluate the effectiveness of the coding scheme in reducing the latency and improving the overall efficiency of the system.

### 4.1. Packet Loss Rate

To analyze the completion probability distribution under varying packet loss rates, we set up the following parameters:

- The initial distance of the source node to the sink node was randomly generated within the range of 0 to 150 m. This was because the current communication range of intelligent cars is 150–300 m. We stipulated that the communication range of the sink node was 150 m in front and behind; that is, the sink node could communicate with vehicles within a distance of 150 m:
- The speed of each node was randomly assigned within a range of 60 to 120 km/h considering the highway scenario;
- The initial position of each car was randomly generated, either in front of or behind the sink node;
- Each time slot was set to a duration of 100 ms, resulting in 10 rounds of communication per second.

With these parameters in place, we calculated the completion probability of the sink node at time slot *t*, which represents the likelihood of receiving *M* packets after *t* time slots.

**Figure 5:**
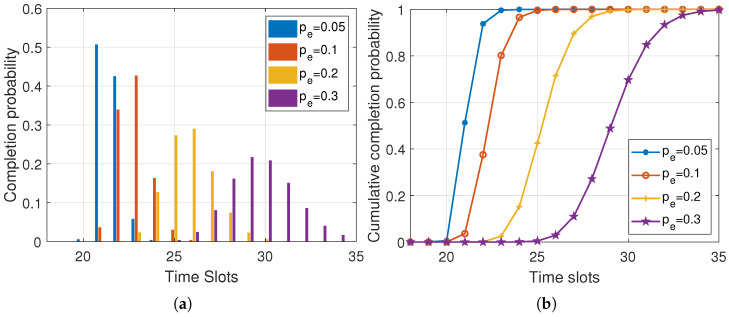
Completion probability distribution and cumulative completion probability distribution of the delay under different packet loss rates (m=2, w=3, M=100). (**a**) Completion probability distribution; (**b**) cumulative completion probability distribution.

Figure 5 shows the results of the simulation conducted with varying packet loss rates of m=2, w=3, and M=100. In Figure 5a, we can observe that as the packet loss rate increased, the sink node required more time to receive *M* data packets, resulting in a more dispersed probability distribution of completion delay. This was because of the decrease in the number of data packets received by the sink node during each round, as a result of the high packet loss rate.Additionally, as the time slots progress, more sources may move out of the sink’s communication range, resulting in fewer sources participating in the communication process and increasing the delay. Figure 5b demonstrates the correlation between the packet loss rate and the slope of the cumulative completion probability distribution curve. As the packet loss rate decreases, the curve becomes steeper, indicating a higher probability of timely completion. This implies that a lower packet loss rate leads to more efficient and reliable completion of the transmission process.

### 4.2. Batch Size

**Figure 6:**
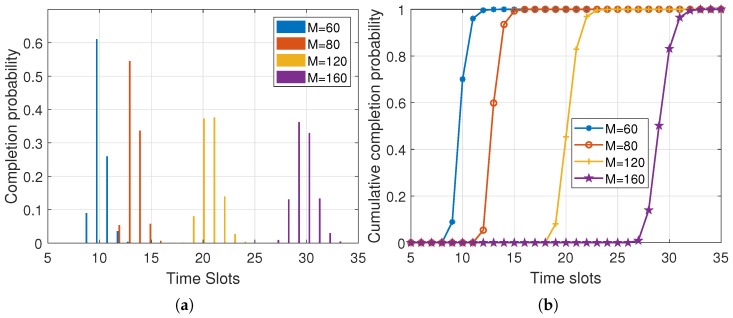
Completion probability distribution and cumulative completion probability distribution of delay under different batch size (m=3, w=4, $p_{e}=0.1$). (**a**) Completion probability distribution; (**b**) cumulative completion probability distribution.

The delay also depends on the batch size. Figure 6 presents the simulation results under different batch sizes, with parameters set as m=3, w=4, $p_{e}=0.1$. In Figure 6a, we can observe the completion probability distribution, and in Figure 6b, we can observe the cumulative distribution of the probability of delay. As the batch size increased, the number of time slots required to transmit a batch also increased. This resulted in a broader probability distribution of completion delay, indicating that larger batch sizes require more time to complete the transmission process. The increased delay was attributed to the larger number of data packets that had to be transmitted within each batch.

Table 2 presents the average delay and unit delay of the first batch of packets transmitted under varying batch sizes. It can be observed that as the batch size increased, the average delay consistently increased. However, contrary to the findings in the one-way lane scenario [18], the unit delay did not always decrease in the two-way lanes scenario. In the two-way lane scenario, after applying RLNC to *M* raw data packets and transmitting them to the sink node, the sink node needed to receive *M* valid data packets to perform decoding. Therefore, with larger batch sizes, more rounds of transmission were required to complete the transmission process, even with the same number of initial sources. Consequently, the duration of the communication process was prolonged, leading to a higher likelihood of source nodes moving out of the communication range of the sink node. Thus, the subsequent rounds witnessed a decrease in the number of sources and the number of packets received in each time slot. It is worth noting that for smaller network sizes, the communication delay became larger, which was due to the limited number of sources available for transmission.

The dynamic nature of the network topology needs to be considered when analyzing the impact of batch size. Merely focusing on the average and unit delay for the first batch is not sufficient. This is because, as subsequent batches are sent, the number of source nodes changes over time, influencing the overall communication delay. Therefore, we analyzed the total delay for sending a total of *Q* data packets, while varying the batch size *M*, under the condition that the total quantity of data packets *Q* remained constant. Table 3 presents the cumulative delay incurred during the transmission of all packets, under varying batch sizes. It can be observed that, as the batch size increased, the total delay exhibited a decreasing trend. This was because, with a smaller batch size, it took more rounds of communication to send the subsequent batches, and the source nodes may have left the communication range, resulting in more time slots. This implies that increasing the batch size can mitigate communication delays and improve the overall efficiency of the transmission process.To reduce the communication delay in two-way lane V2V communication using RLNC, increasing the batch size within the storage and computing capabilities of the source and sink nodes can effectively minimize the communication delay.

### 4.3. Vehicle Range

**Figure 7:**
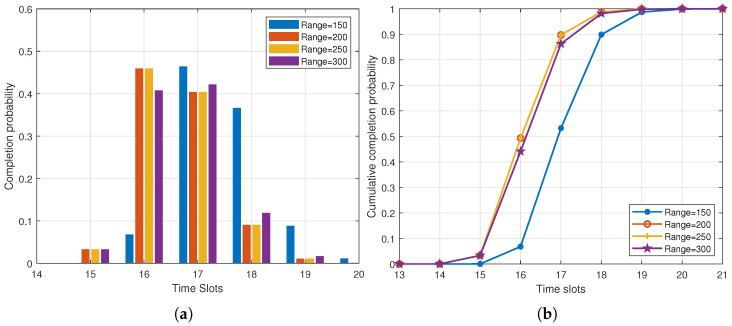
Completion probability distribution and cumulative completion probability distribution of delay under different range of vehicles (m=2, w=3, $p_{e}=0.1$, M=100). (**a**) Completion probability distribution; (**b**) cumulative completion probability distribution.

Next, we examined the influence of varying the vehicle ranges on the time slots required for transmission, as illustrated in Figure 7. Figure 7a shows the completion probability distribution, and Figure 7b shows the cumulative completion probability distribution. It is evident that with a smaller communication range, more time slots were needed to complete the transmission. However, as the communication range increased, there was minimal impact on the transmission process. This was attributed to the fact that with a sufficiently large communication range, vehicles remained within the communication range until the transmission was complete.

### 4.4. Poisson Arrival Process

**Figure 8:**
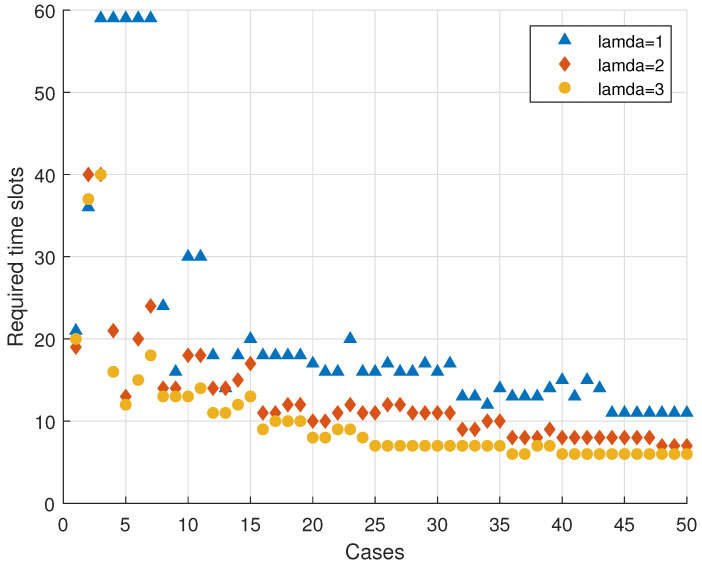
The time slots required with different λ in the Poisson process (m=2, w=3, $p_{e}=0.1$, M=100).

The Poisson process approximates the stochastic arrival process of vehicles well. In the following analysis, we delved into the influence of varying arrival rates on the transmission, utilizing a Poisson process model to simulate vehicle arrivals. As depicted in the Figure 8, we initiated the network with three vehicles in the same direction and four in the opposite direction, assuming a packet loss rate of 0.1. λ represents the Poisson parameter, indicating the arrival rate of vehicles. For each task involving the transmission of 100 data packets, we determined the time slots required for 50 cases. At the beginning of the transmission, there was a period during which the model with λ=1 (represented by blue triangles) required a significantly large number of time slots. This was because the vehicles transmitting initially gradually leave, but due to the relatively low arrival of vehicles, more time slots were needed to complete the transmission.

Notably, after multiple cases, we observed a stabilizing trend in the time required for each task. Once it reached a steady state, a higher vehicle arrival rate (signified by a larger λ) corresponded to a reduced number of time slots being required to complete a transmission. This was because, when there were more vehicles within communication range, more vehicles could participate in the transmission task, enabling faster reception of a sufficient number of decoded data packets.

### 4.5. Coding vs. Non-Coding

By employing RLNC, *M* raw data packets were encoded in batches at the source nodes and transmitted to the sink node, which required the sink node to receive *M* valid data packets for decoding. Through multiple batches, the sink node could recover *Q* original data packets. Without coding, each source node randomly selected and sent one packet to the sink node in each round, until all packets had been received. However, this approach does not guarantee that the randomly selected data packets from different sources will be distinct, potentially leading to the sink node receiving duplicate data packets. Table 4 provides a comparison between the coding and non-coding schemes. It shows that as the packet loss rate increased, the communication latency also increased. However, it is worth noting that doubling the packet loss rate did not result in a significant increase in communication delay. The adoption of RLNC technology improved the communication robustness, enhancing the resistance against channel degradation and reducing communication delay.

In terms of RLNC coding overheads, this hinged on both the finite field size *q* and the number of original packets *N* involved in the coding process. Storing the coefficients in a single coded packet necessitated $\left(N-1\right)\cdot \log_{2}q$ bits. It is evident that, with a small packet size, the performance was significantly hindered due to this substantial overhead. An approach involving the attachment of the seed of the random coefficients generator to the coded packets was employed in [14,15] for network coding, effectively reducing the overheads to $\log_{2}q$, regardless of the number of combined packets. This idea, initially proposed in [25], is worth considering for adoption to reduce overheads in future research.

## 5. Conclusions

In this paper, we proposed an RLNC scheme for efficient and low-latency transmission of large-scale data in V2V communication with two-way lanes. We introduced a dynamic multi-source single-sink model specifically designed for the two-way lane V2V communication scenario and derived the lower bound of the generation probability for the RLNC scheme. Our analysis revealed that reducing the packet loss rate and increasing the sending batch size properly can effectively decrease the communication delay. By conducting a comparative analysis with a non-RLNC scheme, we demonstrated the superior performance of the RLNC scheme in reducing the communication delay and enhancing network robustness in V2V networks with a dynamic topology with two-way lanes.

## Figures and Tables

**Table 1:** Comparative table of our proposed method and recent literature.

	Ours	[21]	[22]	[23]	[24]
With the help of RSU	✗	✓	✗	✓	✓
The level of RLNC	Packet level	Symbol level	Packet level	Packet level	Packet level
One/two-way lane	Two-way lane	One-way lane	One-way lane	Two-way lane	Two-way lane and intersection scenario
Data scale	Large scale	Large scale	Large scale	BSM	BSM

**Table 2:** Unit delay under different batch sizes (in time slots).

$M(m=3,w=4,p_{e}=0.1)$	$E_{T}$	$\bar{T}$
60	10.2545	0.1709
80	13.4206	0.1678
120	20.6697	0.1722
160	29.5586	0.1847

**Table 3:** Total delay under different batch sizes (in time slots).

$M(Q=480,m=3,w=4,p_{e}=0.1)$	$E_{T}$	$M(Q=600,m=4,w=5,p_{e}=0.1)$	$E_{T}$
60	258.0652	75	138.5758
80	255.6062	100	136.7833
120	252.2668	120	136.0340
160	250.0318	150	135.4871

**Table 4:** Comparison of coding and non-coding schemes (in time slots).

Case	$E_{T}\;\left(Q=400,\;M=50,\;m=2,\;w=3\right)$	$E_{T}\;\left(Q=600,\;M=60,\;m=4,\;w=5\right)$
Coding ($p_{e}=0.1$)	270.9284	180.3904
Coding ($p_{e}=0.2$)	327.5296	225.0866
Coding ($p_{e}=0.3$)	399.8219	282.0798
Non-coding ($p_{e}=0$)	514.86	474.73

## Data Availability

Not applicable.
